# Evaluation of the Prognostic Value of Low-Frequency KRAS Mutation Detection in Circulating Tumor DNA of Patients with Metastatic Colorectal Cancer

**DOI:** 10.3390/jpm13071051

**Published:** 2023-06-26

**Authors:** Chien-Yu Lin, Ming-Yin Shen, William Tzu-Liang Chen, Chin-An Yang

**Affiliations:** 1 Integrated Precision Health and Immunodiagnostic Center, Department of Laboratory Medicine, China Medical University Hsinchu Hospital, Zhubei City 302, Taiwan; t55947@mail.cmuhch.org.tw; 2 Department of Colorectal Surgery, China Medical University Hsinchu Hospital, Zhubei City 302, Taiwan; 3 Department of Biomedical Engineering and Environmental Sciences, National Tsing Hua University, Hsinchu 300, Taiwan; 4 College of Medicine, China Medical University, Taichung 404, Taiwan

**Keywords:** circulating tumor DNA (ctDNA), low-frequency KRAS mutation, metastatic colorectal cancer (mCRC), anti-EGFR therapy, next-generation sequencing (NGS)

## Abstract

*KRAS* mutation in tumor tissue is a well-known predictor of resistance to the treatment of anti-EGFR antibodies in metastatic colorectal cancers (mCRC). However, the prognostic value of low-frequency plasma circulating tumor DNA (ctDNA) *KRAS* mutation in predicting treatment resistance in pretreated mCRC patients remains controversial. This study retrospectively reviewed the clinical course, including response to anti-EGFR and anti-VEGF therapies, and changes in serum tumor marker levels along with image studies in mCRC patients with <1.5% *KRAS* mutations detected in plasma ctDNA by next-generation sequencing (NGS) at a single center in Taiwan. We identified six pretreated mCRC patients with low-frequency *KRAS* G12V/G12D/G12S/G13D mutations (variant allele frequency 0.26~1.23%) in plasma ctDNA. Co-occurring low-frequency ctDNA mutations in *APC*, *TP53*, *MAP2K1*, *KEAP1*, or *CTNNB1* were also detected. Although all six patients had treatment adjustments within one month after the ctDNA genetic test, image-evident tumor progression was noted in all patients within a median of 4 months afterwards. Re-challenge therapy with a combination of anti-EGFR, anti-VEGF, and FOLFIRI chemotherapy was found to be ineffective in a patient with 0.38% *KRAS* G12D mutation in baseline ctDNA. Our study suggests that the detection of low-frequency *KRAS* mutations in ctDNA could be used as a predictor of treatment response in mCRC patients.

## 1. Introduction

The detection of *KRAS* mutations in tumor tissue is a well-known predictor of resistance to the treatment of anti-epidermal growth factor receptor (EGFR) antibodies in metastatic colorectal cancer (mCRC) [1]. Together with the location of colorectal cancer, professional guidelines have focused on tumor *KRAS* mutation as a main factor to be considered in treatment choices [2]. While chemotherapy plus an anti-EGFR regimen is recommended for left-sided *RAS* wild-type mCRC, chemotherapy plus anti-VEGF is suggested for the treatment of right-sided *RAS* wild-type or *RAS*-mutant mCRC with the goal of disease control [3]. The addition of anti-EGFR is recommended for the treatment of right-sided *RAS* wild-type mCRC if the goal is a reduction in tumor size [2]. However, whether low-frequency *KRAS* mutation (<5% mutant to wild-type) in tissue might benefit from anti-EGFR therapy remains a controversial issue [1]. It has been reported that colorectal cancer patients with a mutation allele frequency (MAF)/variant allele frequency (VAF) of 0.1~5% in *KRAS* mutation in tumor tissue might benefit from the addition of anti-EGFR (cetuximab) to FOLFIRI chemotherapy [4]. However, another study indicated that colorectal cancer patients with a 2.3~10% *KRAS* mutant allele burden in tumor tissue might be resistant to anti-EGFR therapy, and suggested that a >2.3% MAF detected in tumor tissue should be considered positive for *KRAS* mutation [5]. In terms of the prognostic value of *KRAS* mutation in colorectal cancer, the results from previous studies are also heterogeneous, suggesting that different mutation variants of *KRAS* confer distinct prognoses on the overall survival of colorectal cancer patients [6,7].

Currently, the detection of *KRAS* mutation is routinely carried out in colorectal cancer patients on FFPE tumor tissue. However, it is known that the detection of mutations in circulating tumor DNA (ctDNA) in patients’ plasma not only provides complementary genetic information to tissue tests but may also better reflect real-time tumor heterogeneity in metastatic cancer patients [8]. It has been suggested that a liquid biopsy, such as ctDNA genetic profiling, might have prognostic value for mCRC patients, allowing precise monitoring of treatment response and clonal evolution [9,10]. Both PCR-based methods and next-generation sequencing (NGS) can detect *KRAS* mutation in plasma ctDNA with high sensitivities [11,12]. It is reported that with an input of 20~40 ng ctDNA, the probability of detecting a mutation with MAF of 0.2~0.3% could reach 100% using NGS [13]. Since changes in genetic profiles and percentages of mutant alleles in ctDNA have been reported to serve as an early sign of treatment response or cancer progression [11], we hypothesized that the detection of low-frequency *KRAS* mutations in plasma ctDNA of mCRC patients might have clinical value in disease prognostication and might provide guidance for further decision making.

In our hospital, we established a plasma ctDNA mutation profiling test using NGS for clinical cancer monitoring and actionable drug suggestions according to laboratory guidelines for the interpretation of sequence variants in cancer [14]. This study retrospectively examined the clinical course of six mCRC patients with low-frequency *KRAS* mutation (VAF < 1.5%) detected in plasma ctDNA. We aimed to characterize the heterogeneity of genetic variations accompanying low-frequency *KRAS* mutation detected in plasma ctDNA of these patients, and to evaluate the potential prognostic values of low-frequency ctDNA *KRAS* mutation in cancer progression and treatment responses.

## 2. Methods

### 2.1. Patients

We retrospectively reviewed ctDNA NGS results of mCRC patients screened between May 2021 and April 2023 at China Medical University Hsinchu Hospital. mCRC patients with <1.5% *KRAS* mutation frequencies detected in plasma ctDNA by NGS who followed up at China Medical University Hsinchu Hospital within this period were recruited. Cases of very low *KRAS* mutation frequency (<0.5%) detected in ctDNA were confirmed by real-time PCR (Roche COBAS z480) or a second round of NGS. Analyzed were the clinical course during follow-up, including changes in serum tumor marker levels and image study results, and the concordance of clinical treatment with suggestions of actionable drug information generated by the NAVIFY clinical decision support system (NAVIFY Mutation Profiler, v.2.3.2, Roche, Switzerland). This study was approved by the Institutional Review Board of China Medical University Hospital (CMUH109-REC1-174), and each participant provided written informed consent to participate, in accordance with the Declaration of Helsinki.

### 2.2. Isolation of Plasma ctDNA

Whole blood was collected using Roche Cell-Free DNA Collection Tubes (K3-EDTA; Roche, Switzerland). Plasma was separated by centrifugation at 1600× *g* for 10 min and stored frozen at −70 to −80 °C until ctDNA isolation. For this ctDNA isolation, 4 mL of plasma was thawed and centrifuged at 1600× *g* for 10 min at room temperature, and an Avenio cfDNA extraction kit (Roche, Switzerland) was applied following the manufacturer’s protocol. After extraction, ctDNA concentration was quantified using a QuantiFluor dsDNA System (Promega Corporation, Alcobendas, Madrid, Spain). Additionally, sample quality was assessed using the High-Sensitivity DNA Kit of the Qsep100 system (BIOptic, New Taipei, Taiwan).

### 2.3. Library Preparation and Next-Generation Sequencing

Sequencing libraries were prepared using 20–50 ng ctDNA as sample inputs following the protocol of the AVENIO cfDNA Expanded kit (77 genes, Roche, Switzerland) or the AVENIO cfDNA Surveillance kit (197 genes, Roche, Switzerland). Quality control measures were carried out to assess the concentration and ideal peak size of the enriched library. The ideal peak size was assessed using the Qsep100 system (BIOptic, New Taipei, Taiwan). The expected sequencing library size was more than 300 bp. Sequencing was conducted using the 300-cycle NextSeq 500/550 Mid Output kit v2.5 on the Illumina NextSeq 550 System (Illumina, San Diego, CA, USA).

### 2.4. Data Analysis

Adapter-trimmed FASTQ files were aligned to the hg38 reference genome and variant calling was performed by using the AVENIO Oncology Analysis Software (version 2.0.0). A minimum of 75% of bases generated by each sequencing run were expected to have a base calling quality score (Phred or Q score) range above Q30. Synonymous variants and variants with high population frequencies (according to dbNSFP v.4.3a, gnomAD v.2.1.1, ExAC v.0.3.1) were excluded from further analysis. Variants were then selected by comparison to references in cancer genome/disease databases (COSMIC v. v96, TCGA v.31.0, and ClinVAR v. 20220804). The clinical decision support platform NAVIFY Mutation Profiler v.2.3.2 (Roche, Switzerland) was applied to evaluate the clinical significance and to identify links to actionable therapy options according to medical guidelines.

### 2.5. Validation of Low-Frequency KRAS Mutation in ctDNA

Low-frequency *KRAS* mutations detected in ctDNA by NGS were validated by real-time PCR *KRAS* mutation analysis for ctDNA using the Roche COBAS z480 system and the KRAS Mutation Test v2 LSR kit (Roche, Switzerland) according to the manufacturer’s instructions. Data analysis was performed by uploading files to the online LSR Data Analysis tool (https://lifescience.roche.com/en_nl/brands/oncology-research-kits.html, accessed on 17 March 2023). The lower limit of detection of our plasma ctDNA NGS test for *KRAS* hotspot mutations was 0.17%.

## 3. Results

### 3.1. Patient Characteristics

Six patients (P1~P6) with metastatic CRC (mCRC) and low-frequency *KRAS* mutations (variant allele frequency VAF 0.26~1.23%) detected in plasma ctDNA by our next-generation sequencing (NGS) pipeline were recruited (female *n* = 5, male *n* = 1; age 60.3 ± 12.1 years). Their clinical characteristics, including age, gender, metastatic site, and treatment before receiving the plasma ctDNA test, are shown in Table 1. All patients had received at least one course of chemotherapy with or without targeted therapy (i.e., anti-EGFR, anti-VEGF) before the ctDNA test. P4 and P5 had primary tumors resected before ctDNA testing, and the tumor tissues tested positive for *KRAS* p.G12D and *KRAS* p.G12S, respectively. P2 had a liver metastatic site resected before ctDNA testing, and the metastatic tumor tested negative for *KRAS* mutation. P3 also had a liver metastatic site resected before the ctDNA test, but tissue *KRAS* mutation profiling was not performed. In P6, the primary tumor was resected before the ctDNA test, and tissue *KRAS* mutation profiling was performed on the recurring/more advanced tumor of the primary site noted by image studies 7 months after the ctDNA test, returning a positive test result for *KRAS* p.G12V.

### 3.2. Mutation Profiling of Plasma ctDNA in mCRC

In addition to *KRAS* mutation profiling, we searched for the presence of other oncogenic variants which have been reported in the Catalogue of Somatic Mutations in Cancer (COSMIC), The Cancer Genome Atlas (TCGA), or the ClinVar Database. As shown in Table 2, mutations in *APC* were detected in the plasma ctDNA of three patients, and mutations in *TP53* were detected in the plasma ctDNA of two patients. The presence of *MAP2K1*, *KEAP1*, and *CTNNB1* mutation variants was also detected in plasma ctDNA using NGS panels (Table 2).

**Figure 1:**
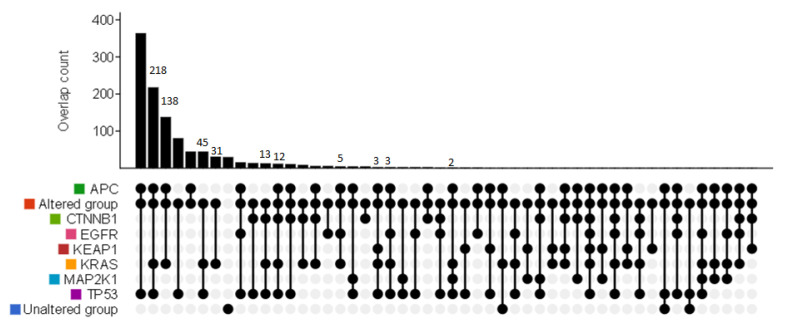
The percentage of mCRC patients of the comparison cBioPortal database cohort with concurrent oncogenic mutations in *KRAS*, *APC*, *TP53*, and other genes in metastatic tumor tissue. Cohort: MSK, Cancer Cell 2018, Patient *n* = 1099. Altered group: patients with mutations, structural variants, or copy number variations in *APC*/*CTNNB1*/*EGFR*/*KEAP1*/*KRAS*/*MAP2K1*/*TP53*.

For comparison, we checked the frequencies of the mutations in the aforementioned genes against those in the resected metastatic colorectal cancer tissues of another mCRC cohort (MSK, Cancer Cell 2018, *n* = 1099) using the cBioPortal database (https://www.cbioportal.org/, accessed on 2 May 2023). The percentages of that cohort’s mCRC patients with concurrent mutations in *KRAS*, *APC*, and *TP53*, concurrent mutations in *KRAS* and *APC*, or concurrent mutations in *KRAS* and *TP53* in tumor tissues were 19.8% (218/1099), 12.6% (138/1099), and 4.1%, respectively (Figure 1). However, data on the corresponding mutations in patients’ plasma ctDNA were unavailable in this cohort.

### 3.3. Analysis of Treatment Suggestions Generated by Decision Support Platform

Next, we analyzed the output suggestions of therapy-related actionable ctDNA mutation variants of each patient using the clinical decision support platform, NAVIFY Mutation Profiler. As shown in Table 3, *KRAS* G12V/G12D/G12S/G13D mutations were suggested to confer resistance to anti-EGFR antibody therapies in colorectal cancer patients. The combination of both *KRAS* and *TP53* mutations was suggested to potentially confer sensitivity to anti-PD1 and CDK4/6 inhibitors. The *KRAS* and *APC* mutation combination was suggested to confer sensitivity to the PI3K and MEK inhibitor combination therapy.

### 3.4. Clinical Course of the Six Patients

**Figure 2:**
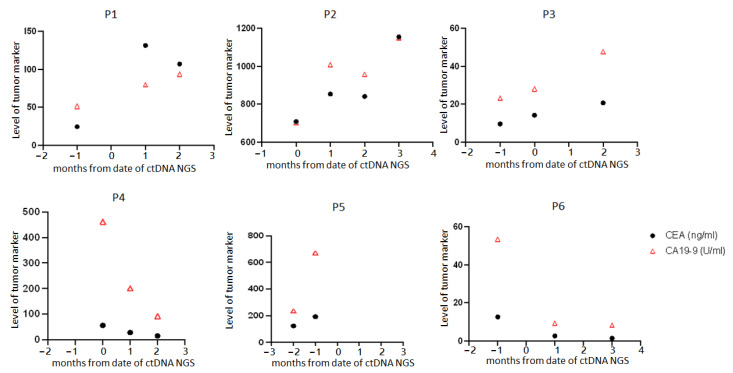
Serial levels of serum CEA and CA19-9 in the 6 patients.

During follow-ups, all six patients had treatment adjustments within 1 month after receiving their plasma ctDNA genetic profiling reports (Table 4). The median time to image (abdominal CT or PET)-evident tumor progression was 4 months (Table 4). The available serial serum CEA and CA19-9 levels before and for the 3 months after the plasma ctDNA mutation test for each patient are presented in Figure 2.

## 4. Discussion

Distinct from tumor tissue *RAS* mutation profiling, it has been reported that ctDNA genetic profiling not only provides real-time information about treatment response but can also indicate minimal residual disease (MRD) and early recurrence levels [15]. In this study, we characterized the heterogeneity of genetic variations accompanying low-frequency *KRAS* mutations detected in plasma ctDNA of six pretreated mCRC patients and analyzed the clinical course of these patients to evaluate the potential prognostic value of low-frequency ctDNA *KRAS* mutations in the differential determination of tumor progression and treatment response.

Of note, the prognostic value of ctDNA on cancer monitoring is dependent on the timing of blood sampling. *KRAS* mutations detected in the ctDNA of colorectal cancer patients sampled at the time of tumor resection might predict a poor response to first-line chemotherapy [16]. In our cohort, P2 and P3 had ctDNA tested more than 3 years after the initial diagnosis because plasma ctDNA mutation profiling was not available in our hospital at that time. However, the detection of ctDNA mutation after resection of the colorectal cancer liver metastatic site and after multiple combination therapies could provide valuable information on the patient’s treatment response and potential mechanisms of resistance. Indeed, *RAS* mutations detected in both tissue and blood ctDNA samples of *RAS-BRAF* wild-type, *HER2*/*MET*-negative mCRC patients have been shown to be major mechanisms of acquired resistance to anti-EGFR therapy [17]. In our study, P1 and P2 with left-sided (sigmoid colon) mCRC had received anti-EGFR (panitumumab/cetuximab) plus chemotherapy before ctDNA testing. Information about tumor *RAS* status was not available for P1, but the metastatic tumor of P2 was tested to be *RAS*-wild-type before chemotherapy, suggesting that the low-frequency *KRAS* mutation detected in P2’s plasma ctDNA almost 6 years after completion of courses of anti-EGFR (cetuximab) + FOLFIRI and anti-VEGF (bevacizumab) + FOLFIRI was a mechanism of acquired resistance. Consistent with a previous report that exposure to EGFR inhibitor was associated with multiple polyclonal mutations in a spectrum of genes [18], we observed concurrent *APC*, *TP53*, and *MAP2K1* mutations besides *KRAS* G12V (VAF 1.04%) in the ctDNA of P1, and concurrent *APC* mutations besides *KRAS* G12D (VAF 0.38%) in the ctDNA of P2.

It has been demonstrated that ctDNA *KRAS* mutations detected in the plasma of mCRC patients 8 weeks after receiving anti-EGFR therapy predict shorter progression-free survival (PFS) when receiving re-challenge therapy with cetuximab and irinotecan as compared to those with wild-type *KRAS* in their plasma ctDNA [19]. Consistent with those results, we found that the levels of CEA and CA19-9 in P2 kept rising 3 months after receiving re-challenge courses with a combination of anti-EGFR (cetuximab), anti-VEGF (bevacizumab), and FOLFIRI chemotherapy, although the proportion of the detected *KRAS* G12D mutation in ctDNA was very low (VAF 0.38%) before re-challenge.

As for the role of *KRAS* mutation in the effectiveness of anti-VEGF therapy in mCRC patients, a study has shown that tumor *KRAS* status could not predict treatment response or affect overall survival (OS) of mCRC patients receiving bevacizumab in combination with oxaliplatin-based regimens [20]. However, another study revealed that mCRC patients harboring specifically *KRAS* G12V/A mutations had inferior OS and PFS when treated with bevacizumab as compared with mCRC patients harboring other *KRAS* mutations [21]. It remains to be determined whether low-frequency *KRAS* G12V/D/S mutations detected in ctDNA confer a poor response in the case of treatment with anti-VEGF in mCRC patients, as seen in P2, P3, and P5 in our study. On the other hand, the clinical decision support system (NAVIFY Mutation Profiler) used in our clinical ctDNA test pipeline suggested potential sensitivity to treatment with a combined regimen of PI3K and MEK inhibitors in CRC patients with *KRAS* G12V mutation or with concurrent *KRAS* and *APC* mutations [22,23]. However, none of the clinicians followed this suggestion in our study, since applying PI3K and MEK inhibitors in treating mCRC patients would be an off-label use and would require further clinical trial investigation.

As shown in our results, we detected the same *KRAS* mutation in both tissue (either from the primary or metastatic site) and plasma ctDNA of the P4, P5, and P6 metastatic rectal cancer patients. In P4 and P5, tumor *KRAS* profiling was performed 1 month before ctDNA testing; therefore, the low-frequency *KRAS* mutation in ctDNA suggests the detection of minimal residual disease levels. In P6, however, tumor *KRAS* profiling was performed 7 months after ctDNA testing at the time of tumor progression. It is likely that the 0.26% *KRAS* G12V mutation detected in the plasma ctDNA of P6 already reflected the presence of the same *KRAS* mutation in tumor tissue at the time of blood sampling, and this mutation contributed to the resistance to anti-EGFR therapy because it was detected 7 months later in the more advanced tumor. These data suggest that the low-frequency *KRAS* mutations detected in ctDNA could provide prognostic information not only about the presence of MRD, but also about the response to anti-EGFR therapy in patients.

Mutations in *KRAS*, *APC*, or *TP53* are frequently found in tumor biopsies of mCRC [24]. Due to the high price, tumor NGS genetic profiling of each patient was not performed in this study. Instead, we analyzed the genetic profiles of resected metastatic tumors of another cohort of mCRC in the cBioportal database and found that mutations in *APC* or *TP53* frequently co-occurred with mutations in *KRAS.* Consistent with that finding, we detected concurrent mutations in *APC* or *TP53* with low-frequency *KRAS* mutation in the ctDNA of four of our six mCRC patients. Co-occurring mutations in *KRAS* and *TP53* have been reported to lead to better response to immune checkpoint inhibitors in non-small-cell lung cancer (NSCLC) [25]. However, co-occurring low-frequency *KRAS* and *TP53* mutations in mCRC might not be associated with increased tumor mutation burden, and thus might not be predictive of treatment response to anti-PD1 therapy. In fact, *KRAS*-mutant CRC has been reported to harbor a more immunosuppressive tumor microenvironment, which limits the use of immune checkpoint inhibitors as monotherapy [7,26].

Our study is limited in that we only sampled plasma ctDNA once for each patient. The VAF of the detected ctDNA *KRAS* mutation might be expected to vary at different time points, and the disappearance or occurrence of other ctDNA mutations be detected in the course of treatment. Longitudinal serial ctDNA gene profiling, together with the evaluation of changes in tumor markers and the results of image studies, would provide a better understanding of MRD, treatment response, and disease prognosis. Our study is also limited because of the small cohort size. In addition, we did not have any patient with low-frequency *KRAS* G12C mutation, a *KRAS* mutation recently identified to be potentially “actionable”, in light of clinical trials using small molecule inhibitors of mutant G12C in combination with cetuximab in heavily pretreated patients with mCRC [27,28].

To sum up, we demonstrated the low-frequency *KRAS* G12V/G12D/G12S/G13D mutations (VAF 0.26~1.23%) detected in plasma ctDNA of pretreated mCRC patients could provide valuable information about MRD and potential resistance to anti-EGFR, and resistance to the combination regimen of anti-VEGF with chemotherapy. Although all patients in our study received changes in clinical treatment within 1 month after ctDNA testing, disease progression was detected in abdominal CT or PET image studies in all cases within a median period of 4 months, suggesting the molecular complexity of mCRC and the importance of considering real-time low-frequency oncogenic mutations in ctDNA as predictors of potential resistance in personalized cancer therapy. Furthermore, we showed that re-challenge therapy with the combination of anti-EGFR, anti-VEGF, and FOLFIRI chemotherapy was ineffective in an mCRC patient with <0.5% *KRAS* G12D mutation in ctDNA.

In conclusion, our study suggests that the detection of low-frequency ctDNA *KRAS* mutations could be used as a predictor of poor treatment response in mCRC patients. Although the development of specific inhibitors targeting all mutant *KRAS* alleles remains challenging, it is an area under vigorous investigation. Recently, the therapeutic efficacy of an inhibitor against KRAS G12D mutated CRC was found to be increased by the combined use of anti-EGFR therapy, which blocked a feedback activation loop driven by EGFR-mediated wild-type RAS signaling [29]. In addition, the detection of the occurrence of low-frequency ctDNA *KRAS* mutation could also hint at the presence of neo-antigen, which makes the application of KRAS mutant-specific CD8+ T cell therapy [30] or the participation in clinical trials using mRNA vaccines encoding novel epitopes for common *KRAS* mutation possible personalized treatment options [7]. Further investigation of larger cohorts of mCRC patients, together with serial ctDNA testing, is required to evaluate the role of low-frequency ctDNA mutations in clinical decision making and cancer monitoring in the era of precision medicine.

## Figures and Tables

**Table 1:** Clinical characteristics of patients.

Patient	Age (Years)	Gender	Primary Location	Metastatic Site	Months since Diagnosis	Surgical Resection before ctDNA Test	Tumor Tissue RAS Mutation	MSS Tumor	Treatment History before ctDNA Test
P1	47	F	Left	Liver	4.5	No	N/A	N/A	Anti-EGFR (panitumumab) + FOLFOX
P2	67	F	Left	Liver, lung, bone	71	Yes (resection of liver metastatic site)	RAS wt	Yes	Anti-VEGF (bevacizumab) + FOLFIRI; Anti-EGFR (cetuximab) + FOLFIRI
P3	69	F	Right	Liver, lung	36	Yes (resection of liver metastatic site)	N/A	Yes	Anti-VEGF (bevacizumab) + FOLFOX; Nivolumab; Anti-VEGFR2 (ramucirumab)
P4	73	F	Rectum	Liver, lung	1	Yes (resection of primary and liver metastatic site)	*KRAS* p.G12D	Yes	FOLFIRI
P5	62	M	Rectum	Liver, lung	1	Yes (resection of primary site)	*KRAS* p.G12S	Yes	FOLFIRI + Oxaliplatin + Anti-VEGF (bevacizumab)
P6	44	F	Rectum	lung	1	Yes (resection of primary site)	*KRAS* p.G12V ^†^	Yes	FOLFOX

MSS: microsatellite stable; N/A: not available; wt: wild-type; FOLFOX: 5-Fluorouracil, Leucovorin, Oxaliplatin; FOLFIRI: 5-Fluorouracil, Leucovorin, Irinotecan. ^†^ Tissue *KRAS* mutation test performed on recurring tumor 7 months after ctDNA test.

**Table 2:** ctDNA mutation profiling.

Patient	KRAS (VAF%)	APC (VAF%)	TP53 (VAF%)	MAP2K1 (VAF%)	KEAP1 (VAF%)	CTNNB1 (VAF%)
P1	p.G12V (1.04%) ^†^	p.E1397* (6.05%)	p.R342* (8.31%)	p.K57T (0.37%), p.L177M (0.38%)		
P2	p.G12D (0.38%) ^‡^	p.R499* (1.24%), p.E1309Dfs*4 (1.60%)				
P3	p.G13D (0.46%) ^†^					
P4	p.G12D (1.23%)	p.R1463X (1.29%)				p.N387K (0.72%)
P5	p.G12S (1.02%)		p.R175H (0.54%), p.C135W (0.38%)		p.T142M (0.39%)	
P6	p.G12V (0.26%) ^†^					

VAF: variant allele frequency. ^†^: ctDNA *KRAS* mutation validated by real-time PCR; ^‡^: ctDNA *KRAS* mutation validated by repeated NGS test. *: a nonsense mutation resulting in a premature stop codon; fs*4: a frameshift mutation caused by deletion of nucleotides, resulting in a new reading frame ending at position 4.

**Table 3:** Treatment recommendations generated by NAVIFY Mutation Profiler for ctDNA mutation profiles detected in patients with MSS colorectal cancers.

Genetic Mutation	Anti-EGFR	Anti-VEGF	Anti-VEGFR	PI3K Inhibitor + MEK Inhibitor	Anti-VEGFR + HDAC Inhibitor	Anti-PD1	CDK4/6 Inhibitor	Anti-EGFR + MEK Inhibitor
KRAS G12V	R (Tier-IA)	R		S				
KRAS G12D/G12S/G13D	R (Tier-IA)			C				
TP53 mutation					S	S		
KRAS + TP53 mutation	R					S	S	
APC mutation								
KRAS +APC mutation				S				
MAP2K1 K57T	R							S
KEAP1 mutation								
CTNNB1 mutation								

R: resistant; S: sensitive; C: controversial.

**Table 4:** Clinical courses of the six patients.

Patient	Change in Clinical Treatment within 1 Month after ctDNA Test	Time to Tumor Size Progression on CT/PET after ctDNA Test (Months)
P1	Add anti-VEGF (bevacizumab)	1.5
P2	Shift to anti-VEGF (bevacizumab) + anti-EGFR (cetuximab) + FOLFIRI	3
P3	Add anti-VEGF (bevacizumab)	6
P4	Shift to FOLFIRI + oxaliplatin	3
P5	Dose titration in FOLFIRI + oxaliplatin	5
P6	Shift to FOLFIRI + anti-EGFR (cetuximab)	7

## Data Availability

The data presented in this study are available in the figures of this manuscript. Raw data can be made available upon request.

## References

[1] Waring P., Tie J., Maru D., Karapetis C.S. (2016). RAS Mutations as Predictive Biomarkers in Clinical Management of Metastatic Colorectal Cancer. Clin. Colorectal Cancer.

[2] Chen H.H., Ke T.W., Huang C.W., Jiang J.K., Chen C.C., Hsieh Y.Y., Teng H.W., Lin B.W., Liang Y.H., Su Y.L. (2021). Taiwan Society of Colon and Rectal Surgeons Consensus on mCRC Treatment. Front. Oncol..

[3] Puccini A., Seeber A., Berger M.D. (2022). Biomarkers in Metastatic Colorectal Cancer: Status Quo and Future Perspective. Cancers.

[4] Van Cutsem E., Lenz H.J., Kohne C.H., Heinemann V., Tejpar S., Melezinek I., Beier F., Stroh C., Rougier P., van Krieken J.H. (2015). Fluorouracil, leucovorin, and irinotecan plus cetuximab treatment and RAS mutations in colorectal cancer. J. Clin. Oncol..

[5] Tougeron D., Lecomte T., Pages J.C., Villalva C., Collin C., Ferru A., Tourani J.M., Silvain C., Levillain P., Karayan-Tapon L. (2013). Effect of low-frequency KRAS mutations on the response to anti-EGFR therapy in metastatic colorectal cancer. Ann. Oncol..

[6] Koulouridi A., Karagianni M., Messaritakis I., Sfakianaki M., Voutsina A., Trypaki M., Bachlitzanaki M., Koustas E., Karamouzis M.V., Ntavatzikos A. (2022). Prognostic Value of KRAS Mutations in Colorectal Cancer Patients. Cancers.

[7] Zhu G., Pei L., Xia H., Tang Q., Bi F. (2021). Role of oncogenic KRAS in the prognosis, diagnosis and treatment of colorectal cancer. Mol. Cancer.

[8] Knebel F.H., Bettoni F., da Fonseca L.G., Camargo A.A., Sabbaga J., Jardim D.L. (2019). Circulating Tumor DNA Detection in the Management of Anti-EGFR Therapy for Advanced Colorectal Cancer. Front. Oncol..

[9] Guttlein L., Luca M.R., Esteso F., Fresno C., Mariani J., Otero Pizarro M., Brest E., Starapoli S., Kreimberg K., Teves P. (2022). Liquid biopsy for KRAS, NRAS and BRAF mutation testing in advanced colorectal cancer patients: The Argentinean experience. Future Oncol..

[10] van ‘t Erve I., Greuter M.J.E., Bolhuis K., Vessies D.C.L., Leal A., Vink G.R., van den Broek D., Velculescu V.E., Punt C.J.A., Meijer G.A. (2020). Diagnostic Strategies toward Clinical Implementation of Liquid Biopsy RAS/BRAF Circulating Tumor DNA Analyses in Patients with Metastatic Colorectal Cancer. J. Mol. Diagn..

[11] Holm M., Andersson E., Osterlund E., Ovissi A., Soveri L.M., Anttonen A.K., Kytola S., Aittomaki K., Osterlund P., Ristimaki A. (2020). Detection of KRAS mutations in liquid biopsies from metastatic colorectal cancer patients using droplet digital PCR, Idylla, and next generation sequencing. PLoS ONE.

[12] Keller L., Belloum Y., Wikman H., Pantel K. (2021). Clinical relevance of blood-based ctDNA analysis: Mutation detection and beyond. Br. J. Cancer.

[13] Heitzer E., Haque I.S., Roberts C.E.S., Speicher M.R. (2019). Current and future perspectives of liquid biopsies in genomics-driven oncology. Nat. Rev. Genet..

[14] Li M.M., Datto M., Duncavage E.J., Kulkarni S., Lindeman N.I., Roy S., Tsimberidou A.M., Vnencak-Jones C.L., Wolff D.J., Younes A. (2017). Standards and Guidelines for the Interpretation and Reporting of Sequence Variants in Cancer: A Joint Consensus Recommendation of the Association for Molecular Pathology, American Society of Clinical Oncology, and College of American Pathologists. J. Mol. Diagn..

[15] Yang W., Zou J., Li Y., Liu R., Yan Z., Chen S., Zhao X., Guo W., Huang M., Li W. (2022). Longitudinal Circulating Tumor DNA Profiling in Metastatic Colorectal Cancer During Anti-EGFR Therapy. Front. Oncol..

[16] Yao J., Zang W., Ge Y., Weygant N., Yu P., Li L., Rao G., Jiang Z., Yan R., He L. (2018). RAS/BRAF Circulating Tumor DNA Mutations as a Predictor of Response to First-Line Chemotherapy in Metastatic Colorectal Cancer Patients. Can J. Gastroenterol. Hepatol..

[17] Pietrantonio F., Vernieri C., Siravegna G., Mennitto A., Berenato R., Perrone F., Gloghini A., Tamborini E., Lonardi S., Morano F. (2017). Heterogeneity of Acquired Resistance to Anti-EGFR Monoclonal Antibodies in Patients with Metastatic Colorectal Cancer. Clin. Cancer Res..

[18] Topham J.T., O’Callaghan C.J., Feilotter H., Kennecke H.F., Lee Y.S., Li W., Banks K.C., Quinn K., Renouf D.J., Jonker D.J. (2023). Circulating Tumor DNA Identifies Diverse Landscape of Acquired Resistance to Anti-Epidermal Growth Factor Receptor Therapy in Metastatic Colorectal Cancer. J. Clin. Oncol..

[19] Cremolini C., Rossini D., Dell’Aquila E., Lonardi S., Conca E., Del Re M., Busico A., Pietrantonio F., Danesi R., Aprile G. (2019). Rechallenge for Patients With RAS and BRAF Wild-Type Metastatic Colorectal Cancer With Acquired Resistance to First-line Cetuximab and Irinotecan: A Phase 2 Single-Arm Clinical Trial. JAMA Oncol..

[20] Kim S.T., Park K.H., Shin S.W., Kim Y.H. (2014). Dose KRAS Mutation Status Affect on the Effect of VEGF Therapy in Metastatic Colon Cancer Patients?. Cancer Res. Treat..

[21] Fiala O., Buchler T., Mohelnikova-Duchonova B., Melichar B., Matejka V.M., Holubec L., Kulhankova J., Bortlicek Z., Bartouskova M., Liska V. (2016). G12V and G12A KRAS mutations are associated with poor outcome in patients with metastatic colorectal cancer treated with bevacizumab. Tumour. Biol..

[22] Zhu M., Jin Q., Xin Y. (2021). Recent clinical advances in PI3K inhibitors on colorectal cancer. Pharmazie.

[23] Gong S., Xu D., Zhu J., Zou F., Peng R. (2018). Efficacy of the MEK Inhibitor Cobimetinib and its Potential Application to Colorectal Cancer Cells. Cell. Physiol. Biochem..

[24] Taghizadeh H., Mader R.M., Mullauer L., Erhart F., Kautzky-Willer A., Prager G.W. (2020). Precision Medicine for the Management of Therapy Refractory Colorectal Cancer. J. Pers. Med..

[25] Dong Z.Y., Zhong W.Z., Zhang X.C., Su J., Xie Z., Liu S.Y., Tu H.Y., Chen H.J., Sun Y.L., Zhou Q. (2017). Potential Predictive Value of TP53 and KRAS Mutation Status for Response to PD-1 Blockade Immunotherapy in Lung Adenocarcinoma. Clin. Cancer Res..

[26] Liu J., Huang X., Liu H., Wei C., Ru H., Qin H., Lai H., Meng Y., Wu G., Xie W. (2021). Immune landscape and prognostic immune-related genes in KRAS-mutant colorectal cancer patients. J. Transl. Med..

[27] Yaeger R., Weiss J., Pelster M.S., Spira A.I., Barve M., Ou S.I., Leal T.A., Bekaii-Saab T.S., Paweletz C.P., Heavey G.A. (2023). Adagrasib with or without Cetuximab in Colorectal Cancer with Mutated KRAS G12C. N. Engl. J. Med..

[28] Pfeiffer P., Qvortrup C. (2022). KRAS(G12C) inhibition in colorectal cancer. Lancet Oncol..

[29] Feng J., Hu Z., Xia X., Liu X., Lian Z., Wang H., Wang L., Wang C., Zhang X., Pang X. (2023). Feedback activation of EGFR/wild-type RAS signaling axis limits KRAS(G12D) inhibitor efficacy in KRAS(G12D)-mutated colorectal cancer. Oncogene.

[30] Tran E., Robbins P.F., Lu Y.C., Prickett T.D., Gartner J.J., Jia L., Pasetto A., Zheng Z., Ray S., Groh E.M. (2016). T-Cell Transfer Therapy Targeting Mutant KRAS in Cancer. N. Engl. J. Med..

